# Neuromorphic NEF-Based Inverse Kinematics and PID Control

**DOI:** 10.3389/fnbot.2021.631159

**Published:** 2021-02-03

**Authors:** Yuval Zaidel, Albert Shalumov, Alex Volinski, Lazar Supic, Elishai Ezra Tsur

**Affiliations:** ^1^Neuro-Biomorphic Engineering Lab, Department of Mathematics and Computer Science, Open University of Israel, Ra'anana, Israel; ^2^Accenture Labs, San Francisco, CA, United States

**Keywords:** neural engineering framework, robotic control software, Loihi, neuromorphic engineering, spiking neural networks, robotic arm

## Abstract

Neuromorphic implementation of robotic control has been shown to outperform conventional control paradigms in terms of robustness to perturbations and adaptation to varying conditions. Two main ingredients of robotics are inverse kinematic and Proportional–Integral–Derivative (PID) control. Inverse kinematics is used to compute an appropriate state in a robot's configuration space, given a target position in task space. PID control applies responsive correction signals to a robot's actuators, allowing it to reach its target accurately. The Neural Engineering Framework (NEF) offers a theoretical framework for a neuromorphic encoding of mathematical constructs with spiking neurons for the implementation of functional large-scale neural networks. In this work, we developed NEF-based neuromorphic algorithms for inverse kinematics and PID control, which we used to manipulate 6 degrees of freedom robotic arm. We used online learning for inverse kinematics and signal integration and differentiation for PID, offering high performing and energy-efficient neuromorphic control. Algorithms were evaluated in simulation as well as on Intel's Loihi neuromorphic hardware.

## Introduction

While computational motion planning and sensing have emerged as focal points for countless state-of-the-art robotic systems, in many ways, they are inadequate when compared with biological systems, particularly in terms of energy efficiency, robustness, versatility, and adaptivity (DeWolf et al., 2016). Consequently, neuromorphic (brain-inspired) computing hardware and algorithms have been used in numerous robotic applications (Krichmar and Wagatsuma, 2011). A typical neuromorphic processor comprises densely connected, physically implemented computing elements that communicate with spikes, and emulate biological neurons' computational principles (Tsur and Rivlin-Etzion, 2020). However, designing algorithms with spiking neurons is a challenging endeavor, as it requires the encoding, decoding, and transformation of mathematical constructs without a central processing unit nor address-based memory. One theoretical framework, which allows for efficient data encoding and decoding with spiking neurons is the Neural Engineering Framework (NEF) (Eliasmith and Anderson, 2003). NEF is one of the most utilized theoretical frameworks in neuromorphic computing, and it was used to design neuromorphic systems capable of perception, memory, and motor control (DeWolf et al., 2020). It serves as the foundation for Nengo, a Python-based “neural compiler,” which translates high-level descriptions to low-level neural models (Bekolay et al., 2014). A version of NEF was compiled to work on the most prominent neuromorphic hardware architectures available, including the TrueNorth (Fischl et al., 2018), developed by IBM research, the Loihi (Lin et al., 2018), developed by Intel Labs, the NeuroGrid (Boahen, 2017), developed at Stanford University and the SpiNNaker (Mundy et al., 2015), developed at the University of Manchester.

Robot state can be defined in configuration space by a set of joint angles defining each limb segment's orientation. Forward Kinematics (FK) refers to the computation used to transform the robot's configuration into its End-Effector's (EE) cartesian coordinates. Inverse Kinematics (IK) refers to the opposite transformation in which a robot's joint configuration is computed from its EE location. While FK can be analytically solved using transformation matrices or trigonometry, IK is usually numerically optimized, as often several joint configurations can produce the same EE position. Many numerical optimization methods were developed for IK, ranging from Jacobian inverse (Lynch, 2017) and fuzzy logic techniques (Hagras, 2004) to artificial neural networks (Koker et al., 2004). Once a target configuration is derived, the robot's actuators are controlled to approach it accurately. The most widely used paradigm for robotic control is to continuously actuate it by minimizing the distance between the robot's current EE location and its designated target. A PID controller applies correction signals based on the error's Proportional, Integral, and Derivative terms (Ang et al., 2005). Robust neuromorphic implementations of IK and PID are an essential milestone for neurorobotics.

In this work, we propose NEF-based neuromorphic algorithms for IK and PID. Algorithms were designed with Nengo and evaluated on both simulation and Intel Loihi neuromorphic hardware (Davies et al., 2018). We used real-time learning and signal integration and differentiation for IK and PID, respectively. Algorithms were utilized for the control of a 6 Degrees of Freedom (DOF) robotic arm. Our implementations offer high performing and energy-efficient neuromorphic robot control, which can be compiled over various neuromorphic hardware. In this work, we evaluated the algorithm performance in simulation and on the Loihi chip.

## Materials and Methods

### Robotic Arm

The robotic arm we used in this research comprises nine servo actuators (7 × Dynamixel's XM540-W270, 2 × Dynamixel's XM430-W350). All actuators are capable of 40 N radial load and have an embedded Cortex-M3 microcontroller. The M3 is coupled with contactless 12 bit absolute encoders, allowing the retrieval of the actuator's position, velocity, and trajectory as feedback for position estimation. The XM540 actuators are used for arm movements and have a stall torque of 10.6 Nm (at 12 v input). The XM430 actuators are used to manipulate the EE (grasping, rotating) and have a stall torque of 4.1 Nm (at 12 v input). Actuators are manufactured by ROBOTIS (Korea). Actuators do not have torque sensors and were therefore actuated by current specifications. The relation between the driven current and the generated rotational velocity is not linear as it has to account for friction. In this work, the current-speed association was estimated as described below. Arm chassis is based on 3D printed grippers (allowing function-tailored customization), ridged and lightweight T-slot extruded aluminum arms, and aluminum brackets. The chassis is connected to the actuators with industrial-grade slewing bearings, and it was assembled by Interbotix (Downers Grove, Illinois). Motion control was evaluated on the Nvidia Xavier chip (Jetson AGX Xavier) and then realized on Intel's Loihi chip. Communication with the daisy-chained servos was based on TTL half-duplex asynchronous serial communication, handled by Dynamixel's U2D2 control board. Overall, the arm design provides 6 DOF, 82 cm reach, 1.64 m span, 1 mm accuracy, and 750 g payload.

### Robot Simulation

To simulate the robot described above, we used the Multi-Joint dynamics with Contact (MuJoCo) physics simulation framework. The robotic arm and joint's accurate dynamic were specified using CAD-derived mechanical description and inertia and mass matrices. CAD and dynamic specifications were provided by Trossen Robotics (USA). The simulation was developed using Nengo, a Python package for building, testing, and deploying NEF-based neural networks.

### Forward and Inverse Kinematics

FK transform a robot's configuration to the cartesian coordinates of its EE. Here, it was implemented using transformation matrices, which characterize the relative transformation (rotation, translation) from each joint to the next. For our five joints robot, FK would take the form of *T* = *T*_01_*T*_12_*T*_23_*T*_34_*T*_45_*T*_56_, where *T*_*ij*_ is the transformation matrix in homogenous coordinates from the reference frame at joint *i* to reference frame at joint *j* (indices 0 and 6 refer to the world's and EE coordinate reference frames, respectively). Initializing *T* with the appropriate set of rotations and translations and multiplying it by a zero vector [0, 0, 0, 1]^*T*^ (in homogenous coordinate) will result in our FK model *T*_*x*_(*q*):

(1) $$\begin{array}{l} {\text{T}}_{\text{x}}\left(q\right)=\left[\begin{array}{c} 0.2\left(s_{4}\left(-c_{3}\left(s_{1}c_{0}c_{2}+s_{2}c_{0}c_{1}\right)+s_{0}s_{3}\right)+c_{4}\left(c_{0}c_{1}c_{2}-s_{1}s_{2}c_{0}\right)\right)+0.3\left(c_{0}c_{1}c_{2}-s_{1}s_{2}c_{0}-s_{1}c_{0}\right)+0.06c_{0}c_{1}\\ 0.2\left(s_{4}c_{3}\left(c_{1}c_{2}-s_{1}s_{2}\right)+c_{4}\left(s_{1}c_{2}-s_{2}c_{1}\right)\right)+0.3\left(s_{1}c_{2}+s_{2}c_{1}+c_{1}\right)+0.06s_{1}+0.118\\ 0.2\left(s_{4}\left(c_{3}\left(s_{1}c_{0}c_{2}+s_{0}s_{2}c_{1}\right)+c_{0}s_{3}\right)+c_{4}\left(s_{0}s_{1}s_{2}-s_{0}c_{1}c_{2}\right)\right)+0.3\left(s_{0}s_{1}s_{2}-s_{0}c_{1}c_{2}+s_{0}s_{1}\right)-0.06s_{0}c_{1} \end{array}\right] \end{array}$$

Where *s*_*x*_ = *sin*(*q*_*x*_), *c*_*x*_ = *cos*(*q*_*x*_), and *q*_*x*_ is actuator *x* angle of rotation. *T*_*x*_(*q*) returns the EE position in the world's coordinate system, where the origin is at the robot's base. The numerical coefficients were derived by calculating the transformations while taking into account the robot's geometry, retrieved from the robot's CAD file.

IK refers to the transformation in which a robot's configuration is computed from its EE's desired location. Generally, IK cannot be analytically solved, and it is, therefore, usually numerically optimized. Here we used the Jacobian inverse for IK. We calculate the Jacobian *J* of *T*, which relates the change of the EE position *x* to the change of joint angles *q*: $J\left(q\right)=\frac{\delta x}{\delta q}$. The Jacobian relates a change in robot configuration $\dot{q}$ to a change in EE position ẋ with:

(2) $$\begin{array}{l} \dot{x\left(q\right)}=J\left(q\right)\dot{q} \end{array}$$

Equation (2) allows us to specify a target in task space—that is, the cartesian space centered on the EE's origin—rather than the space that can be directly controlled, the configuration space. Note that the Jacobian has to be recalculated along the trajectory. With our robotic system, the calculated Jacobian has the shape of (3, 5), where 3 is the number of space dimensions (task space) and 5 is the number of joints (configuration space). To compute IK, we need to invert Equation (2). Since the Jacobian is not necessarily invertible, a common practice is to use its pseudo-inverse form *J*^+^ allowing to compute $\dot{q}=J^{+}ẋ$. Therefore, given an error in space coordinates *x*_*d*_ as the difference between the EE current position *x*_*c*_ and its target position *x*_*y*_, the appropriate change in joint space can be computed using:

(3) $$\begin{array}{l} d\left(q\right)=J^{+}\left(q\right)x_{d} \end{array}$$

Where *d* (*q*) is the change in joint angles, for which the robot's EE will get closer to its target. In each iteration, this equation is re-evaluated until *x*_*d*_ is within some accuracy threshold. Once the joint configuration for a given target point is concluded, control signals, which achieve it, can be calculated using PID control. Further details are provided in Lynch (2017).

### PID Control

PID control is used universally in applications requiring accurate control (Ang et al., 2005). Given a target position, a PID controller will continuously reduce an error signal by providing the robot's actuator with the appropriate control signal to come closer to its target. To do so, the PID controller generates a signal *u* (*t*), which is proportional to the value of the error signal *e* (*t*) (accounting for the current value of the error), to the error integrated value over time (accounting for the *past* values of the error), and to the error derivative (accounting for the projected value of the error), using:

(4) $$\begin{array}{l} u\left(t\right)=K_{p}e\left(t\right)+K_{i}\int_{0}^{t}e\left(t\right)dt+K_{d}\frac{de}{dt} \end{array}$$

Where *K*_*p*_, *K*_*i*_, and *K*_*d*_ are the proportional, integral, and derivative gain coefficients, respectively.

### Neural Engineering Framework

#### Neuromorphic Representation

To represent a computation in a form suitable for neuromorphic hardware, we represent numerical input vectors (or stimuli) with spikes. Stimulus *x* can be represented as *a* using *a* = *f* (*x*), where *a* takes the form of *a* = *G* (*J* (*x*)). *G* is a spiking neuron model and *J* is its input current. Here, we used the leaky-integrate-and-fire (LIF) model (Burkitt, 2006) for *G*. A distributed neuron representation, where each neuron *i* responds independently to *x*, will take the form *a*_*i*_ = *G*_*i*_ (*J*_*i*_ (*x*)). Since neurons usually have some preferred stimuli *e* to which they respond to a high frequency of spikes, *J* can be defined using: *J* = α*x*·*e*+*J*^*bias*^, where α is a gain term, and *J*^*bias*^ is a fixed background current. Note that both *x* and *e* are vectors. Therefore, *x*·*e* equals 1 when both *x* and *e* are in the same direction and 0 when they are opposing each other, where · is the dot product. With NEF, a neuron firing rate δ_*i*_ is defined using (rate coding):

(5) $$\begin{array}{l} \delta_{i}\left(x\right)=G_{i}\left[\alpha_{i}e_{i}\cdot x+{J_{i}}^{bias}\right] \end{array}$$

An ensemble of neurons, in which each neuron has its gain and preferred direction, can distributively represent a high-dimensional stimulus *x*. The represented stimulus $\hat{x}$ (which is an approximation of *x*) can be linearly decoded using:

(6) $$\begin{array}{l} \hat{x}=\underset{i}{\sum }a_{i}\;*\;hd_{i} \end{array}$$

Where *d*_*i*_ are linear decoders, which were optimized to reproduce *x* using least squared optimization and *a*_i_*h is the spiking activity *a*_*i*_ convolved with filter *h* (both are functions of time). Equations (5) and (6) specify the encoding and decoding of mathematical constructs using neuronal ensembles' distributed activity.

#### Neuromorphic Transformation and Online Learning

A key aspect of neuromorphic computing is activity propagation, or the transformation of represented values, implemented by connecting neuron ensembles with a weighted matrix of synaptic connections. The resulting activity transformation is a function of *x*. Notably, it was shown that any function *f* (*x*) could be approximated using some set of decoding weights d^f^ (Eliasmith and Anderson, 2003). Here we will use it to compute Equations (2)–(4) to provide a neuromorphic implementation of IK and PID control. Defining *f* (*x*) in NEF can be made by connecting two neuronal ensembles A and B via synaptic connection weights *w*_*ij*_(*x*) using:

(7) $$\begin{array}{l} w_{ij}=d_{i}⊗e_{j} \end{array}$$

Where *i* is the neuron index in ensemble *A*, *j* is the neuron index in ensemble *B*, *d*_*i*_ are the decoders of ensemble A, *e*_*j*_ are the encoders of ensemble B, which represents *f* (*x*) and ⊗ is the outer product operation.

Connection weights, which govern the transformation between one representation to another, can also be adapted or learned in real-time, rather than optimized during model building. Weights adaptation in real-time is of particular interest in robotics, where unknown perturbations from the environment can affect the error. One efficient way to implement real-time learning with NEF is using the Prescribed Error Sensitivity (PES) learning rule. PES is a biologically plausible supervised learning rule that modifies a connection's decoders *d* to minimize an error signal *e* calculated as the difference between the stimulus and its approximated representation: $\hat{x}-x$. The PES applies the update rule: Δ*d* = κ*eδ*, where κ is the learning rate. Notably, it was shown that when *a* − κ ‖δ‖^2^ (denoted γ) is larger than −1, error *e* goes to 0 exponentially with rate γ. PES is described at length in Voelker (2015).

#### Neuromorphic Dynamical System and Integration

System dynamics is a theoretical framework concerning the non-linear behavior of complex systems over time. Dynamics is the third basic principle of NEF, and it provides the framework with the capacity of using Spiking Neural Networks (SNN) to solve differential equations. It is essentially a combination of the two first principles of NEF: representation and transformation, where we are using transformation in a recurrent scheme. Following Equation (6), a recurrent connection (connecting a neural ensemble back to itself) is defined using: *x* (*t*) = *f* (*x* (*t*)) *h(*t*). A canonical description of a linear error-correcting feedback loop can be described using: $\frac{dx}{dt}=Ax\;(t)+Bu\left(t\right)$, where *x* (*t*) is a state vector, which summarizes the effect of all past inputs, *u* (*t*) is the input vector, *B* is the input matrix, and *A* is the dynamic matrix. In NEF, this standard control can be realized by using:

(8) $$\begin{array}{l} \frac{dx}{dt}=A^{\prime }x\;\left(t\right)+B^{\prime }u\left(t\right) \end{array}$$

Where *A*′ is the recurrent connection, which is defined as τ*A* + *I*, where *I* is the identity matrix, and *B*′ is the input connection, which is defined as τ*B* (Eliasmith and Anderson, 2003). This neural implementation can be used to implement a neuromorphic integrator. For an integrator, input (e.g., velocity) *u* is integrated to define *x* (e.g., position), where ($\frac{dx}{dt}=u$). In terms of Equation (8), *A* = 0 and *B* = 1, resulting in a recurrent connection of *A*′ = 1 and *B* = τ.

### Loihi Chip

In this work, we've implemented IK and PID on Intel's neuromorphic research chip Loihi (Davies et al., 2018). NEF was compiled on the board using the nengo_loihi library (version 0.19) (Lin et al., 2018). The nengo_loihi library was designed to execute Nengo models on Loihi boards. It contains a Loihi emulator backend for rapid model development and a hardware backend for running models on the board itself. Nengo Loihi's hardware backend uses Intel's NxSDK API to interact with the host and configure the board. Each Loihi chip is comprised of 128 neuron-cores; each simulates 1,024 neurons and has 4,096 ports. Each chip also has × 86 cores, which are used for spike routing and monitoring. Communication was established via an SSH channel between our local computer and a virtual machine installed on Intel's neuromorphic research cloud.

## Results

### Simulating Neuromorphic Inverse Kinematics

**Figure 1:**
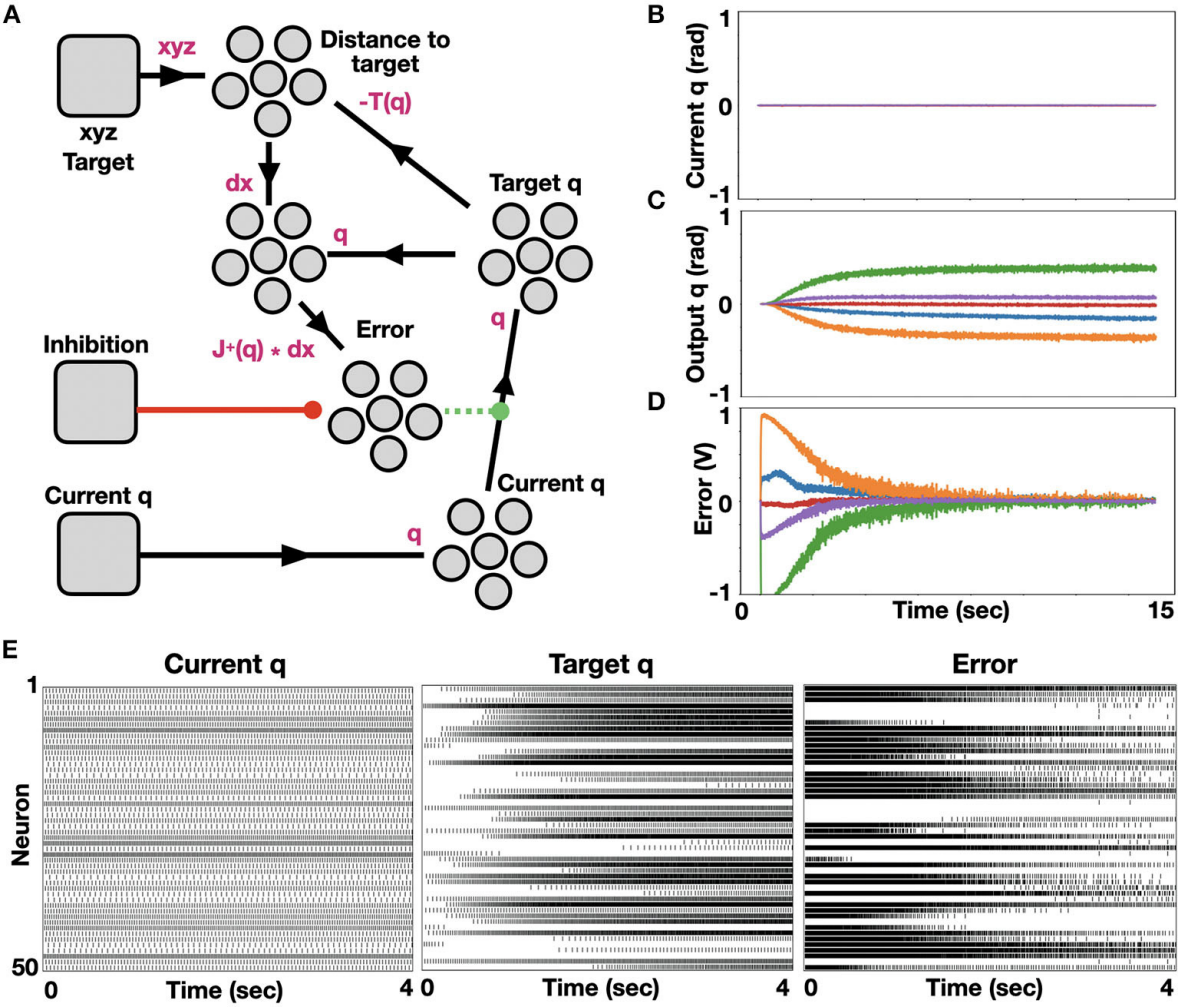
Neuromorphic implementation of IK. **(A)** A model for neuromorphic IK with online learning. Initial joint configuration is represented with neural ensemble Current *q* and transformed to a target joint configuration, represented with neural ensemble *Target q*. This transformation is modulated (or learned) by minimizing an error term, represented with neural ensemble *Error*, defined by distance to target. Nodes, which were used here to introduce signals are shown as rounded squares and ensembles, which represent groups of spiking neurons are represented with a group of 5 circles; **(B)** Initialized zeroed states of the five joints angle; **(C)** Monitored target joint angles as the algorithm optimized arm reaching to point [0.246, 0.62, 0.373] in task space. Each color represents a different joint angle, where bottom to top curves (orange to green) correspond to the base to top joints of the robotic arm; **(D)** Monitored error, demonstrating error flattening as the arm is reaching its target (learning rate is 0.001); **(E)** Raster plots of ensembles *Current_q, Target q* and *Error*.

IK was implemented with NEF using Nengo and tested on our robotic arm. Our model schematic is shown in Figure 1A. In terms of joint angles, the configuration of the robot was introduced through a node into a neuron ensemble denoted *Current q*. Ensemble *Current q* is fully connected to another neuron ensemble, denoted *Target q*. These synaptic weights are modulated to minimize the value decoded from neuron ensemble *Error* using PES optimization. Ensemble *Distance to target* encodes the EE distance from its designated target by subtracting the current position of the EE (calculated using Equation 1, applied on the value decoded from *Target q*) with its desired position (given as an input through node *xyz Target*). Both *Target_q* and the *distance to target* ensembles are connected to a 5D ensemble, allowing for non-linear computation between the five robot's joint states. The difference between the current and the desired robot's joint configuration is calculated through Equation (3), which is implemented through the connection to ensemble *Error*. When the *Error* decoded value is 0, ensemble *Target q* encodes the desired robot configuration. The *Error* ensemble is connected to an *inhibition* signaling node. Upon actuation (initiated once a sufficiently accurate result is achieved), the error signal is zeroed; thus, *Target q* is stabilized at its current state. Here, we performed IK on our robot's 5 joints, starting from an initial configuration where all joints were zeroed (Figure 1B). As learning progresses, the new robot configuration is calculated (Figure 1C), while the error is continually minimized (Figure 1D). Raster plots of ensembles *q, target q*, and *error* are shown in Figure 1E. While the spiking pattern, which represents the initial joint configuration, is constant, the target's spiking pattern changes as the error spiking pattern becomes more amorphous, indicating convergence to zero.

**Figure 2:**
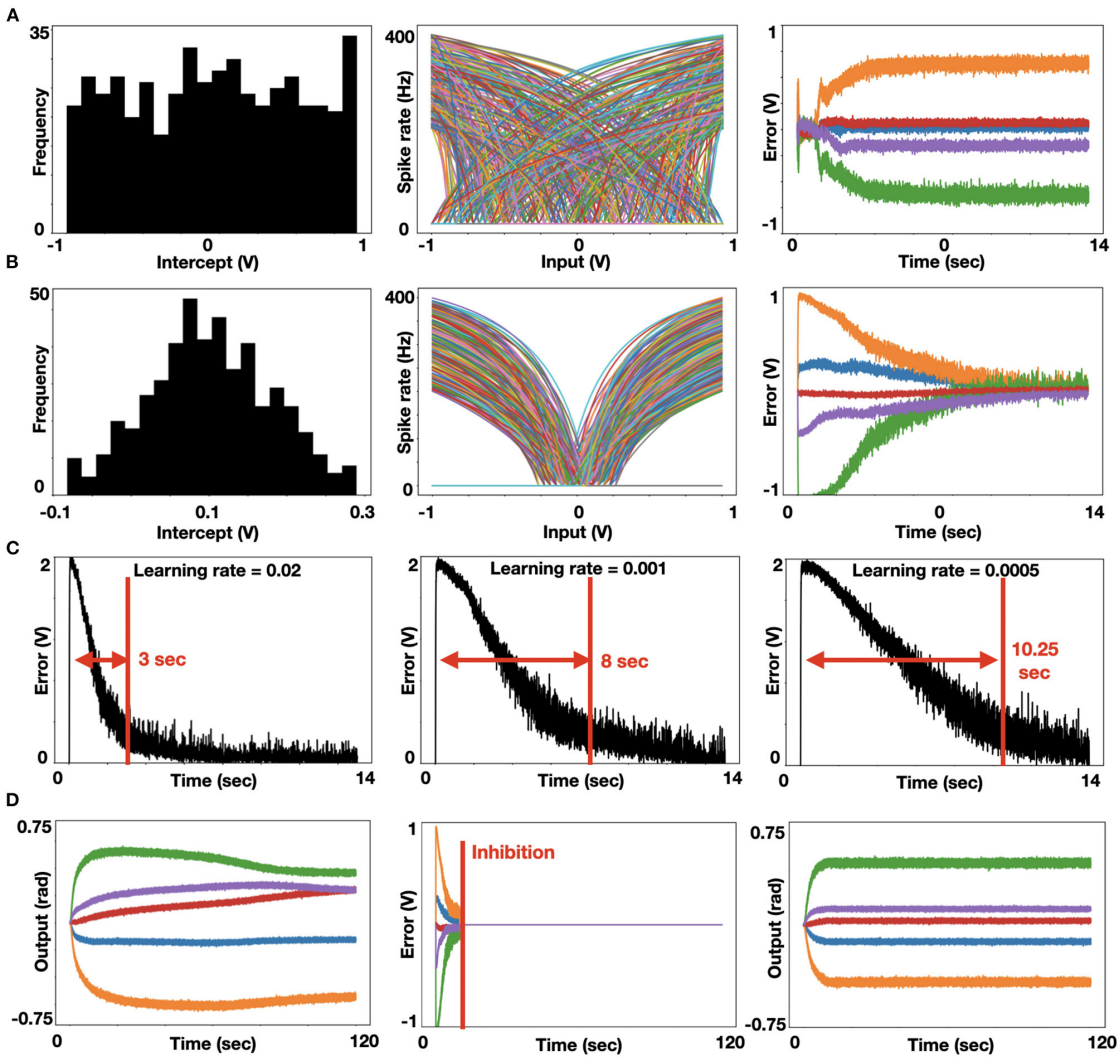
Analysis of neuromorphic IK. **(A)** Histogram of uniformly distributed intercepts (left). The uniformly distributed intercepts as reflected in the tuning curve's distribution, in which spike rate as a function of input is presented for each neuron. Each color represents a different neuron in the ensemble (middle). These uniformly distributed intercepts lead to a non-uniform spanning of the representation space, thus driving non-zero-converging errors. Each color represents a joint's error following the colors' mapping taken in Figure 1 and panel D below (right); **(B)** Histogram of trigonally distributed intercepts (left). Tuning curve's distribution (middle). These triagonally distributed intercepts lead to a uniform spanning of the representation space, thus driving zero-converging errors. Each color represents a joint's error (right); **(C)** Error value for the base joint (indicated otherwise in orange) with three learning rates: 0.02, 0.001, and 0.0005 (left to right), demonstrating that lower learning rates induce slower error convergence. **(D)**. Learned joint configuration with non-inhibited learning leads to a non-stabled joint configuration, as the arm is continuously trying to improve its conformation in space (left). The introduction of an inhibition signal (marked red) is zeroing the error signal (middle). Learned joint configuration with inhibited learning leads to a stable joint configuration (right).

We further analyzed the model by modulating neurons' encoders and learning rates. Each neuron's intercept defines the part of the representation space in which the neuron responds by firing, and it is reflected on the neuron tuning curve (firing rate as a function of input). Note that the intercept is the input value for which the neuron initiates spiking at a high rate. Distributing intercepts uniformly between −1 and 1 makes sense for 1D ensembles for which they create a uniform spanning of that space. This is not the case for high dimensional ensembles. In our implementation, we used high dimensional ensembles to represent the DOF of our robotic system. With uniformly distributed intercepts, the resulted tuning curves are uniformly distributed, resulting in a non-efficient spanning of the representation space. As a result, the system does not converge to its target, as is evident from the error's non-decreasing value (Figure 2A). Changing the intercept distribution to follow a triangular pattern modulates the neurons' tuning curved distribution such that the representation space is adequality spanned. As a result, the system converges to its target, as is evident from the decreasing error (Figure 2B) (Gosmann and Eliasmith, 2016). This modification of the intercept distribution is crucial for accurate representation in 5D space and it is briefly described in DeWolf et al. (2020) and Gosmann and Eliasmith (2016). Our model relies on PES-based optimization, and it is therefore constrained to a prespecified learning rate. We tested our model with three different learning rates, and as expected, error flattening is slower as we increase the learning rate (Figure 2C). Suppose we permit our system to keep optimizing. In that case, the ensembles' fluctuating encoded values induce continuously changing results, where changes in one joint's angle are compensated with changes in other joints (convergence is driven toward a zero gradient potential field). Therefore, we are inhibiting learning once some accuracy threshold is reached, thus holding the computed weights constant and providing a stable target configuration (Figure 2D). Once the desired configuration is calculated, the robot should be actuated accordingly by using, for example, a PID-controller.

### Simulating Neuromorphic PID Controller

**Figure 3:**
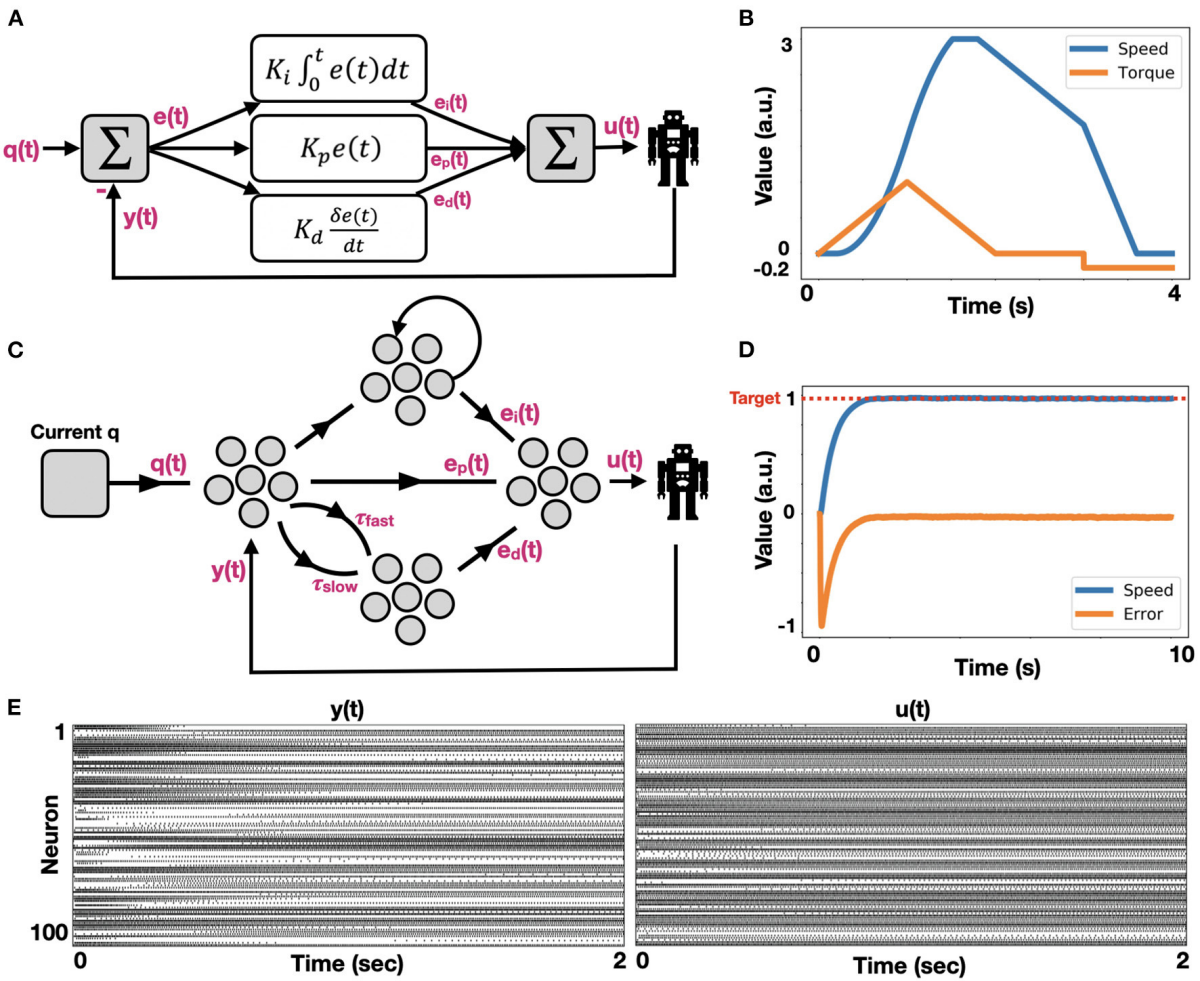
Neuromorphic implementation of PID control. **(A)** The canonical schematic of a PID controller, comprising of integrated [*e*_*i*_(*t*)], proportional [*e*_*p*_(*t*)], and differential [*e*_*d*_(*t*)] error terms; **(B)** Engine actuation model, which is used by our PID controller to induce motion. The engine is induced by a current (blue), generating rotational speed (orange) in a non-linear fashion, as it is taking into account both static and dynamic gear's friction; **(C)** Schematic of a neuromorphic PID controller; **(D)** Neuromorphic PID controller in action. The engine is actuated such that its position is approaching the target while reducing the error; **(E)** Raster plots of ensembles *y* (*t*) (feedback) and *u* (*t*) (robotic control).

A PID controller integrates three error modulations to provide the desired actuation, such that the system would approach a target position. These three signals are described in Equation (4) and are schematically presented in Figure 3A. Our PID controller holds a model of engine actuation. Here, we modeled the actuators using a basic speed-torque (implemented with a driving current) model, which corresponds to our physical actuators. In our model, the actuator experiences static friction and it responds exponentially fast once its gears become active, saturating at some maximum speed. As we stop driving the engine, it is losing momentum due to dynamic friction. When actuation is reversed (current in reversed direction), the position is changed accordingly. The actuation model is shown in Figure 3B. In this work, we implemented the PID with spiking neurons using NEF. The model schematic is shown in Figure 3C. The robot's current configuration is introduced through node *Current q*. We subtract a feedback signal *y* (*t*) from it to compute an error signal. This error signal is propagated to the output ensemble through three paths: 1. Proportional path in which the error is proportionally transformed through a gain factor *k*_*p*_, producing signal *e*_*p*_(*t*); 2. Integration path in which the error is integrated using a neuromorphic integrator (see Methods for further details). The result is scaled by a gain factor *k*_*i*_, producing signal *e*_*i*_ (*t*); and 3. A derivative path, implemented by connecting the error ensemble to a 2D derivative ensemble. To implement derivation, the error is propagated through two synapses: one with a short time scale (τ) and the other with a longer one. The two values are subtracted and scaled by a gain factor *k*_*d*_, producing signal *e*_*d*_ (*t*). These error signals *e*_*p*_(*t*)*, e*_*i*_(*t), e*_*d*_(*t*) are summed in the output ensemble, delivered to the engine as *u* (*t*), which also feedback as *y* (*t*). A running example of this model is shown in Figure 3D. Given a target angle position for the engine (normalized to 1), the error is quickly reduced as the engine's location approaches its target. Raster plots for *y* (*t*) and *u* (*t*) are shown in Figure 3E. These stable spiking dynamics of the control signals reflect fast convergence to target.

**Figure 4:**
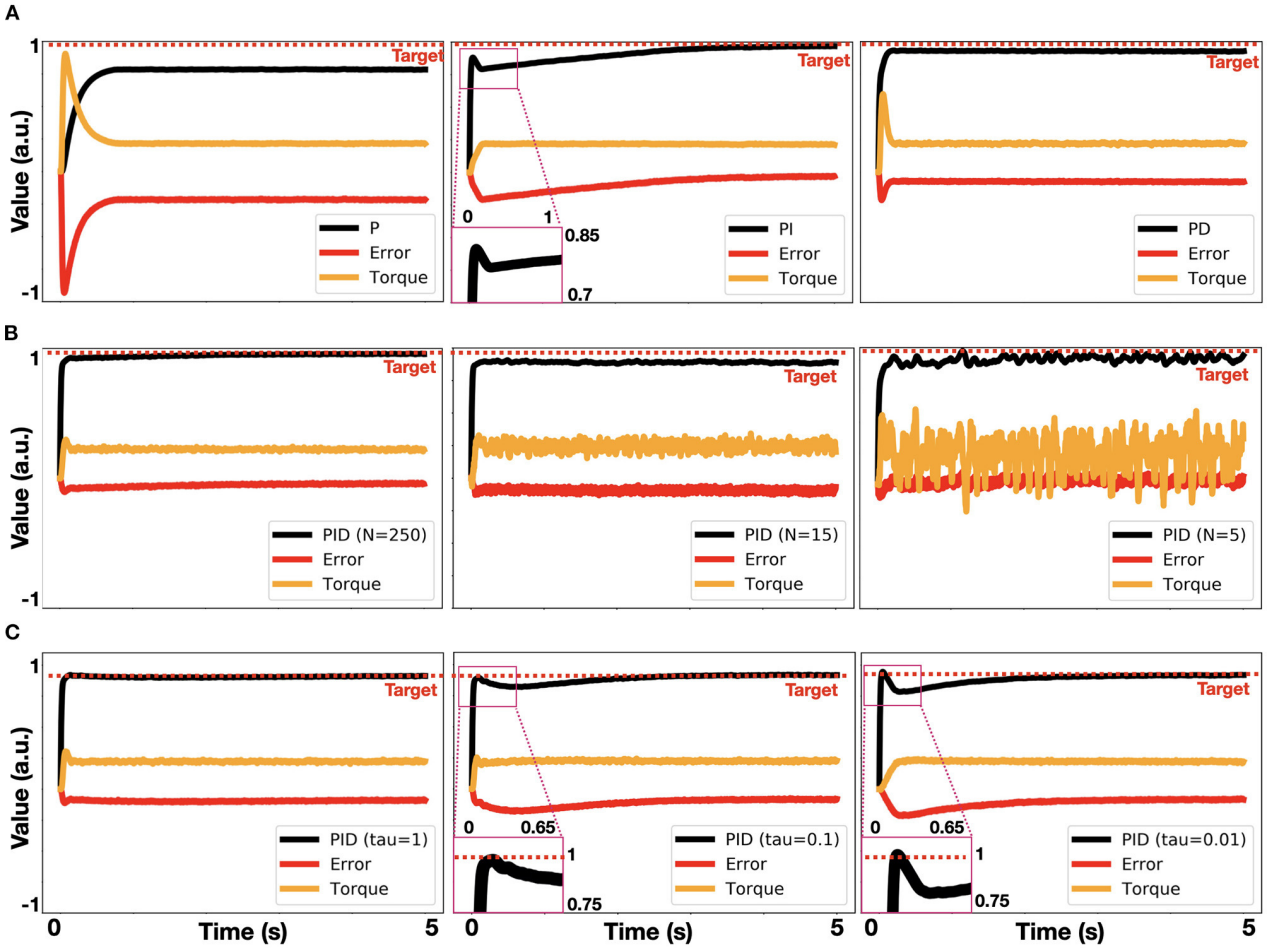
Analysis of neuromorphic PID control. **(A)** Implemented P, PI and PD control (left to right), where τ_*i*_ = 1, τ_*d*_ = 1, *k*_*p*_ = 2, *k*_*i*_ = 1, *k*_*d*_ = 0.4; **(B)** PID control implemented with 250, 15 and 5 neurons per ensemble per dimension (left to right); **(C)** PID control, implemented with τ_*i*_ = 1, 0.1, 0.01 (left to right).

To further analyze our neuromorphic PID control, we examined it as a P (proportional path was enabled), a PI (proportional and integrative paths were enabled), and a PD (proportional and derivative paths were enabled) controller. By implementing all three models, we demonstrated the classic PID characteristics in a neuromorphic implementation. Particularly, the P controller was shown to fall short of reaching the target, the PI controller reached the target with inefficient dynamics, and the PD controller had an improved reaching dynamic, but it failed to reach the target accurately (Figure 4A). We further examined our model by changing the number of neurons. Allocating 250 neurons per ensemble per dimension produced accurate results. Reduced number of allocated neurons dramatically affected performance and stability (Figure 4B). This result is compatible with the noise characteristics of NEF-based representation in which the decoders-induced static noise is proportional to $\frac{1}{N^{2}}$, where *N* is the number of neurons (Eliasmith and Anderson, 2003). Synaptic time constants also constrain neuromorphic implementations. Reducing these time-constants inhibits the integration dynamic (Equation 8), as demonstrated in Figure 4C.

### Robotic Control

**Figure 5:**
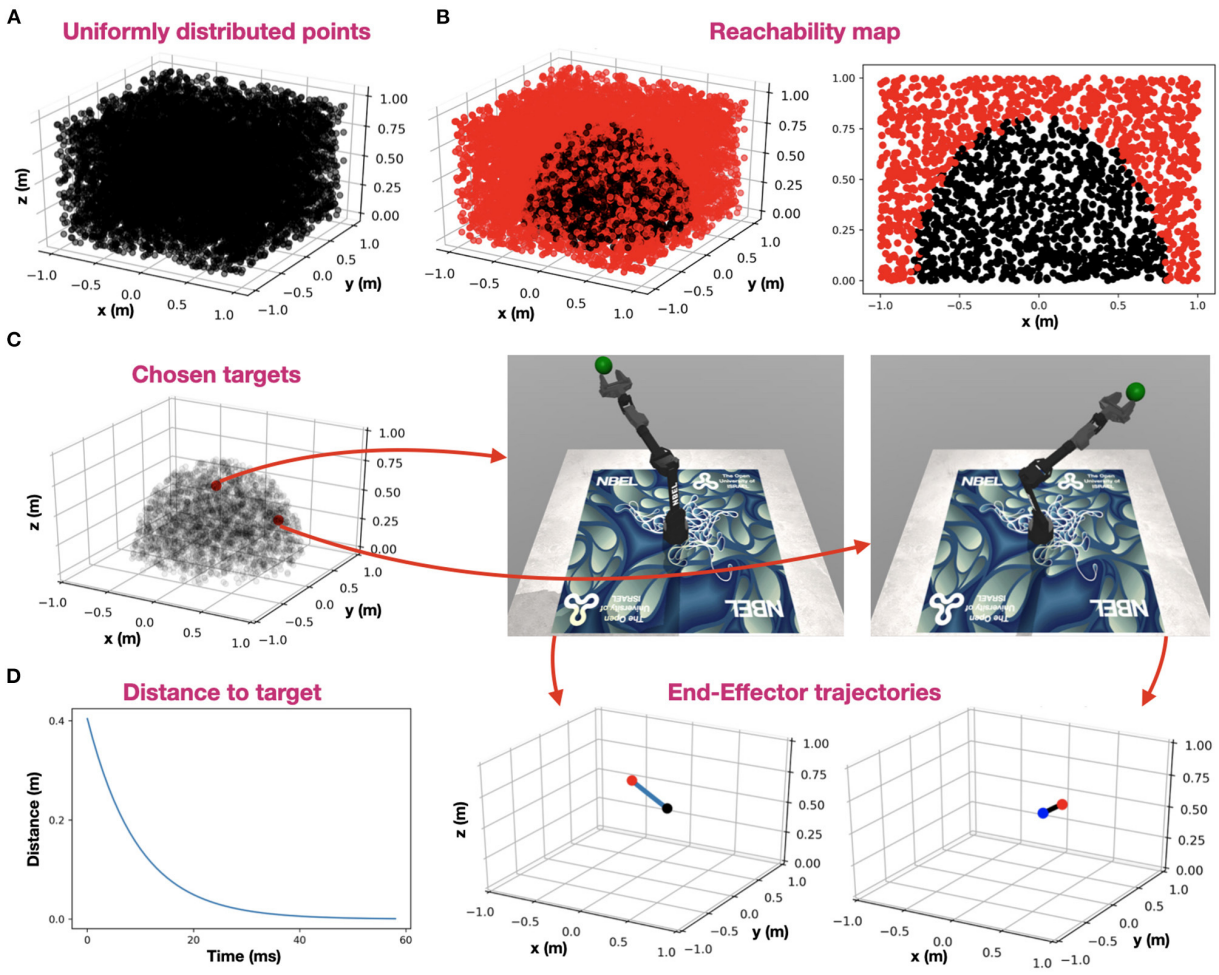
Robot control. **(A)** uniformly distributed 10,000 target points in a 2 × 2 × 1 meters volume; **(B)** IK reachability map, where a black point designates a reachable point with an accuracy of at least 1 mm and a red point designates a non-reachable point. Arm base is located at the origin (0, 0, 0). A 3D reachability map is shown in the left panel, and a cross-section at y = 0 is shown in the right panel; **(C)** Two random points (red) were chosen among the reachable points (semi-transparent black). The arm configuration, resolved for each of the two target points are shown on the right, and the corresponding EE trajectories are shown on the bottom (target is indicated with a red point, EE-origin with a black point and trajectory in blue); **(D)** Distance to the furthest target while reaching it using PID control.

Control was evaluated on a physical 6 DOF robotic arm (described in the Methods). To demonstrate robot performance, we utilized the MuJoCo physical simulator, in which we accurately described the dynamic and mechanical characteristics of our physical arm. Here, we used it to demonstrate the integration of neuromorphic IK and PID control in a physical setting. While IK was used to derive the robot configuration from the desired EE location in space, PID was used to actuate the robot, generating an EE trajectory toward the target. We created a uniformly distributed 10,000 target points in a 2 × 2 × 1 meters volume (Figure 5A). We tested each point for reachability using IK, constructing a 3D reachability map, where a black point designates a reachable point with an accuracy of at least 1 mm (Figure 5B). Arm base is located at the origin (0, 0, 0). For demonstration, we randomly chose two points and used PID control to generate robot motion. The selected points, the final arm configuration, and the generated EE trajectories are shown in Figure 5C. Trajectories are linear (minimal path) as expected. Distance to target curve is shown in Figure 5D, demonstrating fast convergence to target.

### Loihi Implementation

**Figure 6:**
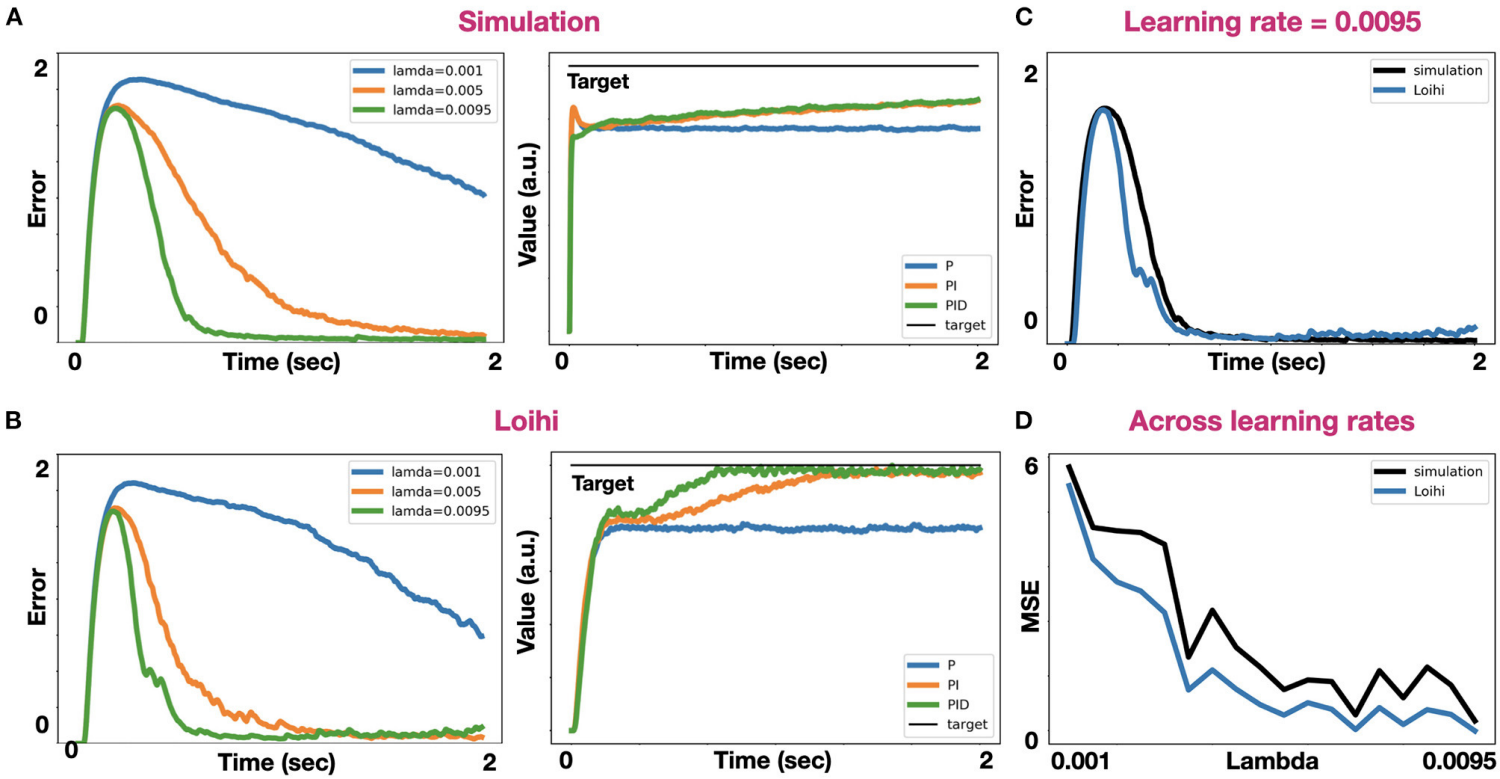
Comparative analysis between simulation and the Loihi. **(A)** IK error trace in simulation with three different learning rates: 0.001, 0.005, and 0.0095 (left). PID-control in a simulation where *k*_*p*_= 1, *k*_*i*_ = 0.1, *k*_*d*_= 0.35, and τ = 0.1 (right); **(B)** IK error trace (left) and PID control (right) on the Loihi, with the same parameters used in simulation; **(C)** Super imposed IK error traces on simulation and the Loihi with a learning rate of 0.0095; **(D)** Mean squared error for IK across different learning rates on both simulation and the Loihi.

We implemented both IK and PID control on Intel's Loihi chip. When implementing IK with different learning rates, the same error convergence pattern appears in both simulation and on the board (Figures 6A,B). However, superimposed results showed that the Loihi could converge better, as its error reduced faster than in the simulated model (Figure 6C). We found it to be consistent for various learning rates (Figure 6D). When implementing PID control on the Loihi, we found it hard to implement the derivative pathway, as it is currently cannot support high synaptic time constants. Defining a long time-constant is essential, as the generated control signal fluctuates, affecting our capacity to derive the signal's rate of change accurately. However, for a time-constant of 10 ms, and a configuration of *k*_*p*_= −1, *k*_*i*_ = −0.1, *k*_*d*_= 0.35, the Loihi was able to converge faster than simulation to the desired target, pointing out its embedded learning accelerator (Davies et al., 2018).

## Discussion

IK and PID control are two of the most fundamental algorithms for robotic control. While IK allows for defining trajectories in task space and implementing it in configuration space, PID provides a canonical way of efficiently approaching a target. Neuromorphic control algorithms may acquire some of the advantages of biological motor control. These neuromorphic algorithms may closely emulate key features of neurophysiological analogs, such as cerebro-cerebellar inverse models (Ishikawa et al., 2016), in the case of IK, and vestibular, oculomotor circuits (Lenz et al., 2008), in the case of PID control. Notably, the cerebellum is known for maintaining internal forward and inverse models for motion control. Moreover, it was shown that the vestibulo-ocular reflex integrates inertial and proportional visual information to drive the eyes in the opposite direction to head motion, achieving retinal image stabilization. However, from a pure engineering perspective, executing control models with energy-efficient hardware is an important endeavor, regardless of its biological plausibility. For example, SpikeProp is one of the most widely utilized back-propagation-based learning rules for SNNs (Bohte et al., 2000), regardless of backpropagation being biologically plausible or not (Lillicrap et al., 2020).

The notion of utilizing artificial neural networks for inverse kinematics and robot control was explored back in 1993 (Jack et al., 1993) and more recently revisited by Csiszar et al. (2017). Neuromorphic implementations, which are based on SNN, have gained tremendous traction in past decay due to the increased attention to neurorobotics and, more recently, the emergent availability of neuromorphic software and hardware frameworks. Accordingly, neuromorphic implementation of IK and PID control was addressed in several studies. For example, Folgheraiter et al. (2019) utilized LIF neurons to implement a learning algorithm for adaptive motion control. Barhen and Gulati (1991) demonstrated neuromorphic inverse kinematics, concentrating on redundant manipulators, using terminal attractors. More recently, PID controllers have been neuromorphically implemented on an FPGA board by Linares-Barranco et al. (2020) and on the Loihi chip by Stagsted et al. (2020). Interestingly, Tieck et al. (2009) demonstrated a neuromorphic PID-based control with no need for inverse kinematic nor planning. These approaches, however, are hardware/software – framework specific. NEF has the advantage of being able to deploy on numerous neuromorphic hardware. IK with NEF was demonstrated by DeWolf et al. in their work on the REACH adaptive controller (DeWolf et al., 2016) and, more recently, in DeWolf et al. (2020). REACH uses adaptive signals computed online (using PES-learning) to modulate arm movement to adapt to unexpected conditions. Our implementation takes a more direct approach, aiming specifically at neuromorphic IK by transforming task space to configuration space with a single adjustable connection.

Neuromorphic systems are fundamentally limited to the number of neurons, the encoding error, and the synaptic time constants. In this work, we addressed these constraints in the context of robotic control. NEF-based representation is limited to a distortion error, which is induced by the decoders themselves. Representation error is expressed with:

(9) $$\begin{array}{l} E=\frac{1}{2}\int_{-1}^{1}{\left(x-\bar{x}\right)}^{2}dx=\frac{1}{2}\int_{-1}^{1}{\left(x-\overset{n}{\underset{0}{\sum }}a_{i}d_{i}\right)}^{2}dx \end{array}$$

Where *x* is the encoded stimulus, $\bar{x}$ is the represented stimulus, *a*_*i*_ is the activity of neuron *i*, *n* is the number of neurons, and *d*_*i*_ are the computed decoders, derived by the minimization of *E*. This static distortion is proportional to the number of neurons, according to $E\;\approx \;\frac{1}{n^{2}}$ (Eliasmith and Anderson, 2003). As we increase the number of neurons, representation error is reduced (Figure 4B). However, there is much more to it. The selection of the encoders and the distribution of the neurons' tuning curves (intercept, maximal firing rate) have a drastic effect on the representation, especially in higher dimensions. Distributing intercepts uniformly between −1 and 1 makes sense for 1D ensembles. However, in a uniformly occupied 2D space, a neuron with an intercept of 0.75 fires spikes for only 7.2% of the represented space. In higher dimensions, the proportions become exponentially smaller (or larger for negatively encoded neurons). In high dimensions, the naive distribution of intercepts results in many neurons that rarely produce spikes or are always active, providing a poor representation (see Figure 2A). A rational distribution of encoders, particularly choosing encoders following a triangular distribution (DeWolf et al., 2020), dramatically improved the representation as was demonstrated in Figure 2B. Our design is also constrained to synaptic time constants, which govern the PID's integral and derivative pathways, and the learning rate, which regulates the learning pace of the IK model. The time constant constraints on the PID's integrative path were explored in Figure 4C, and the effect of the learning rate on the IK model was demonstrated in Figure 2C. While in simulations, these time constants can be arbitrarily defined to range across time scales, and indeed biological counterparts to these signals extend from just a few milliseconds to minutes and hours, current neuromorphic hardware does not provide the same flexibility. This fact might have a dramatic effect when a derivate of a noisy signal has to be calculated. The Loihi chip, for example, only supports a time constant of up to 100 ms. Working with such short time constants forces a more accurate representation. However, implementing the model on the Loihi suggests that its embedded learning circuitry (Davies et al., 2018) allows it to converge faster to the target compared to the simulated model (Figure 6B). The Loihi representation accuracy is also demonstrated in the IK model, where it performed continuously better than the simulated model across different learning rates (Figure 6D).

In this article, we presented SNNs capable of PID control and learning-based IK. We explored their implementation on both simulation and neuromorphic hardware, thus demonstrating NEF-based models for neurorobotics. Our implementations use neuromorphic learning for IK and signal integration and differentiation for PID, offering high performing and energy-efficient robotic neuromorphic control.

## Data Availability Statement

The original contributions presented in the study are included in the article, further inquiries can be directed to the corresponding author.

## Author Contributions

YZ and AS designed, implemented the algorithms, and analyzed the results. LS and AV contributed to the discussions and revised the manuscript. EE conceptualized the research, designed the algorithms, and wrote the manuscript. All authors contributed to the article and approved the submitted version.

## Conflict of Interest

LS is employed by Accenture Labs (San Francisco, USA). The remaining authors declare that the research was conducted in the absence of any commercial or financial relationships that could be construed as a potential conflict of interest.
